# Question formulation skills training using a novel rubric with first-year medical students

**DOI:** 10.5195/jmla.2021.935

**Published:** 2021-01-01

**Authors:** Jonathan Eldredge, Melissa A. Schiff, Jens O. Langsjoen, Roger N. Jerabek

**Affiliations:** 1 jeldredge@salud.unm.edu, Associate Professor, Health Sciences Library and Informatics Center, Family & Community Medicine Department, School of Medicine, College of Population Health, University of New Mexico, Albuquerque, NM; 2 mschiff@salud.unm.edu, Research Professor, Division of Epidemiology, Biostatistics, and Preventive Medicine, Department of Internal Medicine, University of New Mexico, Albuquerque, NM; 3 jlangsjoen@salud.unm.edu, Associate Professor, Division of Hospital Medicine, Department of Internal Medicine, University of New Mexico, Albuquerque, NM; 4 rjerabek@salud.unm.edu, Associate Scientist II, Program, Evaluation, Education, and Research, School of Medicine, University of New Mexico, Albuquerque, NM

## Abstract

**Objective:**

The authors used an assessment rubric to measure medical students' improvement in question formulation skills following a brief evidence-based practice (EBP) training session conducted by a health sciences librarian.

**Method:**

In a quasi-experimental designed study, students were assessed using a rubric on their pre-instructional skills in formulating answerable EBP questions, based on a clinical scenario. Following their training, they were assessed using the same scenario and rubric. Student pre- and post-test scores were compared using a paired *t*-test.

**Results:**

Students demonstrated statistically significant improvement in their question formulation skills on their post-instructional assessments. The average score for students on the pre-test was 45.5 (SD 11.1) and the average score on the post-test was 65.6 (SD 5.4) with an average increase of 20.1 points on the 70-point scale, *p*<0.001.

**Conclusion:**

The brief instructional session aided by the rubric improved students' performance in question formulation skills.

## INTRODUCTION

The pivotal first step in the evidence-based practice (EBP) process hinges on formulating an answerable question. A focused question will guide one efficiently to finding the needed evidence. Conversely, a poorly constructed question will misdirect a search for the needed evidence, thereby wasting precious time. As two EBP pioneers once observed, “fuzzy questions tend to lead to fuzzy answers” [1].

EBP can be defined as “a way of providing health care that is guided by a thoughtful integration of the best available scientific knowledge with clinical expertise” [2]. EBP serves as a decision-making framework in clinical practice. The “evidence” in EBP can include original research articles, point-of-care resources, diagnostic tests, physical exams, or patient histories [3]. After the first step of formulating an answerable question, steps two through four consist of searching for the evidence, critically appraising the evidence, and making a decision on applying the evidence to the patient. A fifth, more reflective step, involves evaluating one's performance.

EBP has had a deep and sustained influence on the health professions, and most medical school curricula now include teaching EBP [4]. The approaches employed for teaching EBP are diverse [5, 6]. EBP textbooks stress the importance of clearly stated questions for correctly launching the EBP process and offer practical advice to learners. Health sciences librarians frequently teach medical students how to compose questions in the context of their EBP coursework [7]. Librarians are natural collaborators in teaching EBP question formulation skills, having worked for well over a century in the broader area of question formulation [8–10].

EBP instructors commonly employ a structure for formulating questions that consists of patient, intervention, comparison, and outcome (PICO) elements. The patient element might include some clinically relevant demographic characteristic like the patient's age and pertinent medical problems; intervention consists of the treatment under consideration; comparison relates to the control treatment; and outcome pertains to results of clinical importance [11, 12]. The widely cited Fresno test [13–16] of evidence-based medicine [17] that assesses learners' EBP skills and knowledge incorporates PICO, albeit for only two brief segments. The Berlin questionnaire for evaluating learned EBP skills models the PICO structure in a similar way [18], and an assessment tool by Wyer et al. also uses PICO [19]. Some practitioners have suggested alternatives to PICO. For example, the SPIDER question formulation format relates to qualitative instead of quantitative EBP studies [20]. Davies inventoried an impressive array of alternatives to PICO, including some cited in this article, although these alternatives focus primarily upon evidence-based library and information practice rather than the broader health care professions context of EBP [21].

A growing body of evidence points to the limitations to PICO despite its common use. A team of early EBP physicians created the PICO structure in 1995 with no known input by health sciences librarians or other information professionals [11]. An exploratory study by Huang et al. compared actual clinicians' questions with PICO and found PICO did not represent those questions well. Importantly, they also determined that PICO was most suitable for treatment EBP questions, rather than all other types of EBP questions [22]. However, only roughly half of EBP questions relate to treatment [23, 24], so the PICO format may not adapt well to diagnosis, prognosis, epidemiology, or other types of EBP questions. Schardt et al. found no statistical difference between using and not using PICO search templates for searching in PubMed [25], whereas Hoogendam et al. found PICO to be deficient for launching a timed PubMed search [26]. Furthermore, a 2018 review of whether PICO improved the quality of searches in a variety of databases proved inconclusive [27].

A Cochrane Collaboration–sponsored systematic review on interventions to teach learners how to formulate questions underscored the importance of the topic, stating that “formulating questions is fundamental to the daily life of a healthcare worker” [28]. The systematic review reported that PICO question formulation interventions, most involving residents or practicing clinicians while excluding medical students, produced mixed results. The authors of the Cochrane systematic review selected only four randomized controlled trials on question formulation. These four key studies employed different educational interventions and units of measurement, had methodological issues, and/or produced inconclusive results [29–32].

The Cochrane systematic review, plus the authors' observations of students regularly struggling with the PICO format for many of the same reasons reported in the literature, provided the rationale for improving instruction on question formulation skills at our medical school. Therefore, we evaluated the effect of a new approach to training first-year medical students on question formulation that included a brief instructional session and a novel rubric intended to overcome perceived limitations of existing approaches.

## METHODS

### Population

We performed a quasi-experiment pre-/post-test study [33, 34] with 107 first-year medical students who were enrolled in a required “Quantitative Medicine One” course at the University of New Mexico's School of Medicine to assess the impact of the new EBP instructional approach on formulating an EBP question. This required course incorporated biostatistics, epidemiology, and EBP topics. The course began during the sixth month of the first year of medical school. None of the 107 students had previously learned EBP question formulation skills. The first 3 authors—consisting of a faculty librarian/informatacist, physician epidemiologist, and hospitalist—served as the faculty instructors for the course. The University of New Mexico Institutional Review Board approved this study (19-008).

### Instructional intervention

All 107 students took a pre-test to gauge their pre-instruction skills in formulating questions on their first day of class. The interventional instruction occurred 28 days later, and the post-test occurred within 30 hours after the intervention.

The pre-test presented students on the first day of the course with a case vignette of an elderly patient with Parkinson's disease:

You are at a rural site in your Doctoring 3 (“PIE”) experience during the summer. Today, you are enjoying the work, even though you miss your friends back at medical school. Manual Garcia, age seventy-three, is in the clinic. During the last two months Mr. Garcia has experienced recurring leg tremors, complaints of “weakness,” apathy, slowness in his movements, unilateral rigidity, shuffling gait, and instability when walking. Your preceptor is seeing him today about Mr. Garcia's recent fall in his kitchen. Mr. Garcia appears to be fine, yet shaken from the fall. Your preceptor has diagnosed Mr. Garcia as having fairly advanced-stage Parkinson's disease. You know about Parkinson's disease based on your having taken the “Neurosciences Block” earlier this year. The discussion expands to include possible drugs that might improve the quality of life for Mr. Garcia. Your preceptor discusses possibly prescribing levodopa or a dopamine agonist.

Formulate a question based on this clinical vignette that, when answered, will lead to the best treatment of this patient.

The same 107 students received training on question formulation for 25 minutes as part of an overview of the EBP process in an active learning, large studio classroom setting. At the beginning of the large group session, students were asked, “Why do you think that formulating answerable questions will be important for your individual professional education and for your career?” Small groups consisting of about 8 students, seated at tables, listed their answers to this question on whiteboards. The groups reported their rationales, which the faculty librarian tied together for real-time thematic analysis. He described a systematic review on questions raised by clinicians at the point of care [35], stressing the possible number of questions raised per patient as a means of underscoring the importance of question formulation skills.

About halfway through this large group session, the faculty librarian introduced the rubric (Table 1), as previous studies suggested that students were more likely to accept a rubric in the earlier, formative stages of learning a new skill [36]. The interactive mode of instruction offered practical tips for using the rubric as a checklist. The twenty-five-minute segment of class on question formulation guided students through the steps of focusing on the problem or disease, amplifying the critical details, and composing a free-standing question as guided by the rubric. The faculty librarian gave them examples of well-formulated questions composed by medical students in previous years to facilitate self-confidence among the current students and contrasted these model questions with less productive questions. Students first worked alone and then with a partner in their small groups in a pair-share team to formulate their own diagnosis, treatment, or prognosis types of EBP questions.

**Table 1:** Rubric for evaluating formulated evidence-based practice (EBP) questions

Element	Points
Identifies and focuses upon the main problem or disease	10
Minimizes “noise” in formulated question by removing unneeded elements	10
Amplifies the signal in the question, *as applicable*, with:
Semantic qualifiers (examples: acute/chronic, insidious/abrupt, proximal/distal, sharp/dull)	5
Scale (examples: neoplastic staging, child development Tanner stages)	5
Temporality (examples: duration of illness, length of treatment, seasonality, etc.)	3
Describes the population aspects (age, geography, ethnicity, income)	7
Question composition:
Composes question clearly so a targeted answer can be pursued	5
Question accurately reflects contextual details	5
The final formulated question “stands by itself”	15
Possible categorizations (if applicable):
Identifies question as diagnosis, treatment, or prognosis type	5
Total points	70

Note: Full points are given if the item does not apply to the clinical vignette or question prompt because there would have been no way to select a correct item response.

Within thirty hours of the large group session, all students participated in labs consisting of twenty-five to thirty students in which the faculty librarian briefly showed them examples of diagnosis, treatment, and prognosis EBP questions and fielded any questions. He then gave them five minutes to formulate an answerable question based on the same clinical vignette as that in the pre-test. Students were allowed to use their rubrics as checklists during the post-test. The remainder of the session revolved around searching for evidence, primarily in PubMed. The instructors scored the pre- and post-tests using the rubric to measure the degree of student improvement, with the post-test results passed along to the students before they completed a similar but graded assignment on question formulation without the clinical vignette prompt. Later in the course, the instructors briefly introduced the PICO format to familiarize students with it, in case they encounter it later in their careers.

### The rubric

We scored students' formulated questions using the rubric (Table 1). The prototype of the current rubric was initially developed in 2013 through a series of trial and error approaches involving first- and second-year medical students. It was improved iteratively across 2014–2018 through formal and informal feedback from students and faculty.

The current version of the rubric features three major sections. The first section evaluates students' *focus* on the main problem or disease. This focus on the disease or problem prepares the learner to translate this concept to effective literature searching by finding the appropriate Medical Subject Heading (MeSH) in PubMed that best represents the problem or disease. The second section evaluates students' *amplification of* essential clinical details, such as descriptive adjectives (semantic qualifiers), scales, temporality, or population. These adjectives can alert the practitioner about which studies to include or exclude in matching the evidence to the specific patient, and details concerning the population can inform the search by including or excluding certain populations, using filters related to age groups, and using key MeSH terms, such as those related to socioeconomic factors, class, risk factors, or protective factors. The third section determines if the learner has *composed* a question that a reader can understand without the reader knowing the broader clinical context. In other words, “the question stands by itself.”

### Statistical analysis

Students' mean pre- and post-test scores were analyzed with a paired t-test using Stata, version 15 (College Station, TX).

## RESULTS

The 107 students who underwent the brief training and use of the rubric improved their question formulation skills by a statistically significant margin. The average score for students on the pre-test was 46 (standard deviation [SD] 11, 95% confidence interval [CI] 43–48), whereas the average score on the post-test was 66 (SD 5, 95% CI 65–67). Students did not perform well on the pre-test, as expected, largely because approximately half of the students did not mention the disease.

## DISCUSSION

We found that question formulation skills taught using this new instructional intervention and rubric yielded a significant improvement of 20.1 points on average on a 70-point scale. Those who have taught students, residents, and practitioners know that teaching others how to formulate answerable questions can be far more challenging than it might first appear to be. Question formulation expects the learner to initially use inductive logic and then transition to using deductive logic [37–39]. The wording of a question also can introduce unintended ambiguity and confusion [40, 41].

This study differed from previous library and EBP studies on question formulation in several important ways. Our medical students were new to EBP question formulation, whereas populations in other studies likely had prior question formulation training, particularly with PICO. Second, we used a new approach to question formulation to overcome perceived limitations of the PICO structure. Third, the rubric evolved in a collaborative context involving librarian, basic sciences, and clinical faculty members. Fourth, this new approach and rubric easily accommodated diagnosis, prognosis, and epidemiology as well as treatment types of EBP questions.

The interval of no more than thirty hours between the intervention and the post-test minimized the likelihood of biases such as regression to the mean or secular changes [42]. This brief interval also prevented threats to internal validity such as the historical artifact or maturation [43–45]. The faculty librarian used a near identical approach in teaching question formulation and the same rubric with physician assistant and medical residents during most of these same years, 2014–2019. Learners in these other education programs demonstrated similar improvements in formulating questions, although this study with medical students represented the first rigorous research study to measure improved student performance.

As this study involved medical students at one specific medical school, it likely has limited generalizability. The same instructional protocols and rubric have been used at our medical school and other degree programs at our institution for several years with similar outcomes however, so our results appear to remain consistent and, thereby, reliable in this institution.

Although we might have preferred using an experimental design, this curriculum posed too many opportunities for contamination, because students belong to several concurrent academic groups during the first two years at this medical school. This multiple group membership would place intervention and control students in daily contact, thereby risking contamination of the control participants by the intervention participants. This study could not measure students' long-term retention of question formulation skills because the curricular schedule scattered students among clinical assignments following the course. Furthermore, the study design did not allow a distinction between the separate effects of the teaching and rubric.

Future studies are needed to replicate these results, and multicenter studies could greatly extend their generalizability. It would be interesting to measure the effect of the instruction alone and rubric alone in isolation from one another, although this experiment would involve considerable logistic obstacles due to possible contamination of controls. Furthermore, as the faculty librarian created the rubric to improve subsequent searches, future studies could seek to demonstrate a connection between question formulation and search quality.

This prospective quasi-experiment involving pre- and post-test scores demonstrated that a brief instructional session in question formulation skills accompanied by a new rubric improves students' skills. Question formulation skills help students become lifelong learners and more immediately appear to improve their critical thinking skills [46]. This new approach places greater emphasis on the needed elements for question formulation than the popular Fresno test. Also, the instructional session improves upon the PICO approach by expanding its versatility beyond treatment types of EBP questions to include epidemiology, diagnosis, risk, harm, and prognosis types of questions. Therefore, this study might signal a way forward to diversify how trainers teach EBP question formulation skills.

## Data Availability

De-identified score data are available in the University of New Mexico's Digital Repository at https://digitalrepository.unm.edu/hsc_hslic/1/.

## References

[1] Oxman AD, Guyatt GH Guidelines for reading literature reviews. CMAJ. 1988 4 15;138(8):697–703. 3355948 PMC1267776

[2] National Institutes of Health, National Library of Medicine. Medical subject headings (MeSH) [Internet] The Institutes [cited 30 Apr 2020]. <https://www.ncbi.nlm.nih.gov/mesh?otool=unmlib>.

[3] Guyatt G, Rennie D, Meade M, Cook D, eds; American Medical Association. Users' guides to the medical literature: a manual for evidence-based clinical practice 3rd ed. New York, NY: McGraw-Hill Education; 2015.

[4] Liaison Committee on Medical Education. Functions and structure of a medical school: critical judgment/problem-solving skills: standard 7.4 [Internet] Chicago, IL: The Committee; 2020 [cited 14 May 2020]. <https://lcme.org/publications/>.

[5] Nicholson J, Spak JM, Kovar-Gough I, Lorbeer ER, Adams NE Entrustable professional activity 7: opportunities to collaborate on evidence-based medicine teaching and assessment of medical students. BMC Med Educ. 2019 9 3;19(1):330 DOI: 10.1186/s12909-019-1764-y. 31481060 PMC6724374

[6] Maggio LA, Tannery NH, Chen HC, ten Cate O, O'Brien B Evidence-based medicine training in undergraduate medical education: a review and critique of the literature published 2006–2011. Acad Med. 2013 7;88(7):1022–8. DOI: 10.1097/ACM.0b013e3182951959. 23702528

[7] Maggio LA, ten Cate O, Chen HC, Irby DM, O'Brien BC Challenges to learning evidence-based medicine and educational approaches to meet these challenges: a qualitative study of selected EBP curricula in U.S. and Canadian medical schools. Acad Med. 2016 1;91(1):101–6. 26200580 10.1097/ACM.0000000000000814

[8] Taylor RS Question-negotiation and information seeking in libraries. Coll Res Libr. 1968 5;29(3):178–94.

[9] American Library Association. The reference interview: connecting in person and in cyberspace. presentations and responses from the RUSA president's program, 2002 ALA Annual Conference, Atlanta, June 17, 2002 Ref User Serv Q. 2003;43(1):37–51.

[10] Ross CS The reference interview: why it needs to be used in every (well, almost every) reference transaction. Ref User Serv Q. 2003 Fall;43(1):38–43.

[11] Richardson WS, Wilson MC, Nishikawa J, Hayward RS The well-built clinical question: a key to evidence-based decisions. ACP J Club. 1995 Nov-Dec;123(3):A12–3. 10.7326/ACPJC-1995-123-3-A12 7582737

[12] Straus SE, Glasziou P, Richardson WS, Haynes RB Evidence-based medicine: how to practice and teach EBM 5th ed. Edinburgh, UK: Elsevier; 2019 p. 22.

[13] Ramos KD, Schafer S, Tracz SM Validation of the Fresno test of competence in evidence based medicine. BMJ. 2003 2 8;326(7384):319–21. 12574047 10.1136/bmj.326.7384.319 PMC143529

[14] McCluskey A, Bishop B The adapted Fresno test of competence in evidence-based practice. J Contin Educ Health Prof. 2009 Spring;29(2):119–26. 19530195 10.1002/chp.20021

[15] Thomas RE, Kreptul D Systematic review of evidence-based medicine tests for family physician residents. Fam Med. 2015 2;47(2):101–17. 25646982

[16] Shaneyfelt T, Baum KD, Bell D, Feldstein D, Houston TK, Kaatz S, Whelan C, Green M Instruments for evaluating education in evidence-based practice: a systematic review. JAMA. 2006 9 6;296(9):1116–27. 16954491 10.1001/jama.296.9.1116

[17] University of California San Francisco, Family Practice Residency Program. Fresno test of evidence based medicine. grading rubric (form A) Fresno, CA: The University.

[18] Fritsche L, Greenhalgh T, Falck-Ytter Y, Neumayer HH, Kunz R Do short courses in evidence based medicine improve knowledge and skills? validation of Berlin questionnaire and before and after study of courses in evidence based medicine. BMJ. 2002 12 7;325(7376):1338–41. 12468485 10.1136/bmj.325.7376.1338 PMC137813

[19] Wyer PC, Naqvi Z, Dayan PS, Celentano JJ, Eskin B, Graham MJ Do workshops in evidence-based practice equip participants to identify and answer questions requiring consideration of clinical research? a diagnostic skill assessment. Adv Health Sci Educ Theory Pract. 2009 10;14(4):515–33. 18766450 10.1007/s10459-008-9135-1

[20] Cooke A, Smith D, Booth A Beyond PICO: the SPIDER tool for qualitative evidence synthesis. Qual Health Res. 2012 10;22(10):1435–43. DOI: 10.1177/1049732312452938 Epub: 24 7 2012. 22829486

[21] Davies KS Formulating the evidence based practice question: a review of the frameworks. Evid Based Libr Inf Pract. 2011 6 24;6(2):75–80. DOI: 10.18438/B8WS5N.

[22] Huang X, Lin J, Demner-Fushman D Evaluation of PICO as a knowledge representation for clinical questions. AMIA Annu Symp Proc. 2006:359–63. PMC1839740 17238363

[23] Ely JW, Osheroff JA, Ebell MH, Bergus GR, Levy BT, Chambliss ML, Evans ER Analysis of questions asked by family physicians regarding patient care. West J Med. 2000 5;172(5):315–9. 18751285 10.1136/ewjm.172.5.315 PMC1070879

[24] Bjerre LM, Paterson NR, McGowan J, Hogg W, Campbell CM, Viner G, Archibald D What do primary care practitioners want to know? a content analysis of questions asked at the point of care. J Contin Educ Health Prof. 2013 Fall;33(4):224–34. 24347101 10.1002/chp.21191

[25] Schardt C, Adams MB, Owens T, Keitz S, Fontelo P Utilization of the PICO framework to improve searching PubMed for clinical questions. BMC Med Inform Decis Mak. 2007 6 15;7:16. 17573961 10.1186/1472-6947-7-16 PMC1904193

[26] Hoogendam A, de Vries Robbé PF, Overbeke AJPM Comparing patient characteristics, type of intervention, control, and outcome (PICO) queries with unguided searching: a randomized controlled crossover trial. J Med Libr Assoc. 2012 4;100(2):121–6. DOI: 10.3163/1536-5050.100.2.010. 22514508 PMC3324808

[27] Eriksen MB, Frandsen TF The impact of patient, intervention, comparison, outcome (PICO) as a search strategy tool on literature search quality: a systematic review. J Med Libr Assoc. 2018 10;106(4):420–31. DOI: 10.5195/jmla.2018.345. 30271283 PMC6148624

[28] Horsley T, O'Neill J, McGowan J, Perrier L, Kane G, Campbell C Interventions to improve question formulation in professional practice and self-directed learning. Cochrane Database Syst Rev. 2010 5 12;(5):CD007335 DOI: 10.1002/14651858.CD007335.pub2. 20464753 PMC13290143

[29] Bradley DR, Rana GK, Martin PW, Schumacher RE Real-time, evidence-based medicine instruction: a randomized controlled trial in a neonatal intensive care unit. J Med Libr Assoc. 2002 4;90(2):194–201. 11999177 PMC100764

[30] Villanueva EV, Burrows EA, Fennessy PA, Rajendran M, Anderson JN Improving question formulation for use in evidence appraisal in a tertiary care setting: a randomised controlled trial [ISRCTN66375463]. BMC Med Inform Decis Mak. 2001;1:4 Epub: 8 11 2001. 11716797 10.1186/1472-6947-1-4 PMC59901

[31] Schaafsma F, Hulshof C, de Boer A, van Dijk F Effectiveness and efficiency of a literature search strategy to answer questions on the etiology of occupational diseases: a controlled trial. Int Arch Occup Environ Health. 2007 1;80(3):239–47. Epub: 21 6 2006. 16944192 10.1007/s00420-006-0126-3

[32] Cheng GY Educational workshop improved information-seeking skills, knowledge, attitudes and the search outcome of hospital clinicians: a randomised controlled trial. Health Info Libr J. 2003 6;20(suppl 1):22–33. 10.1046/j.1365-2532.20.s1.5.x 12757433

[33] Shadish WR, Cook TD, Campbell DT Experimental and quasi-experimental designs for generalized causal inference Belmont, CA: Wadsworth; 2011 p. 103–15.

[34] Shek TS, Wu J Quasi-experimental designs In: Frey B, ed. The SAGE encyclopedia of educational research, measurement, and evaluation. Vol. 4 Thousand Oaks, CA: SAGE Publications; 2018 DOI: 10.4135/9781506326139.

[35] Del Fiol G, Workman TE, Gorman PN Clinical questions raised by clinicians at the point of care: a systematic review. JAMA Intern Med. 2014 5;174(5):710–8. DOI: 10.1001/jamainternmed.2014.368. 24663331

[36] Reddy YM, Andrade H A review of rubric use in higher education. Assess Eval High Educ. 2010 7;35(4):435–48.

[37] Becker HS Tricks of the trade Chicago, IL: University of Chicago Press; 1998 p. 194–212.

[38] Browne MN, Keeley SM Asking the right questions: a guide to critical thinking Upper Saddle River, NJ: Prentice-Hall Press; 2004.

[39] Babbie E Practice of social research Belmont, CA: Wadsworth Cengage Learning; 2013 p. 54–6.

[40] Education section - what does the question mean? J Evid Based Med. 2014 2;7(1):65–6. 25155571 10.1111/jebm.12089

[41] Education section - what does the question mean, continued. J Evid Based Med. 2014 5;7(2):151. 25155773 10.1111/jebm.12106

[42] Willett WC Nutritional epidemiology In: Rothman KJ, Greenland S, Lash TL Modern epidemiology 3rd ed. Philadelphia, PA: Lippincott Williams & Wilkins; 2008: 582.

[43] Kerlinger FN Foundations of behavioral research 2nd ed. New York, NY: Holt, Rinehart and Winston; 1973 p. 262–3.

[44] Campbell DT, Stanley JC Experimental and quasi-experimental designs for research Chicago, IL: Rand McNally College Publishing Company; 1963 p. 5–6.

[45] Creswell JW Research design: qualitative, quantitative, and mixed methods approaches 3rd ed. Thousand Oaks, CA: Sage Publications; 2009 p. 162–6.

[46] Wang J, Wang D, Chen Y, Zhou Q, Xie H, Chen J, Li Y The effect of an evidence-based medicine course on medical student critical thinking. J Evid Based Med. 2017 11;10(4):287–92. DOI: 10.1111/jebm.12254 Epub 2017 5 24. 28452179

